# Impact of Denoising on Deep-Learning-Based Automatic Segmentation Framework for Breast Cancer Radiotherapy Planning

**DOI:** 10.3390/cancers14153581

**Published:** 2022-07-22

**Authors:** Jung Ho Im, Ik Jae Lee, Yeonho Choi, Jiwon Sung, Jin Sook Ha, Ho Lee

**Affiliations:** 1 CHA Bundang Medical Center, Department of Radiation Oncology, CHA University School of Medicine, Seongnam 13496, Korea; junghosta@naver.com; 2 Department of Radiation Oncology, Yonsei University College of Medicine, Seoul 03722, Korea; ikjae412@yuhs.ac (I.J.L.); pokemon30@yuhs.ac (J.S.); 3 Department of Radiation Oncology, Gangnam Severance Hospital, Seoul 06273, Korea; yunhoc12@yuhs.ac (Y.C.); hjshjs83@yuhs.ac (J.S.H.)

**Keywords:** radiation therapy, contouring, organs at risk, deep-learning-based auto-segmentation, denoiser

## Abstract

**Simple Summary:**

We investigated the contouring data of organs at risk from 40 patients with breast cancer who underwent radiotherapy. The performance of denoising-based auto-segmentation was compared with manual segmentation and conventional deep-learning-based auto-segmentation without denoising. Denoising-based auto-segmentation achieved superior segmentation accuracy on the liver compared with AccuContour^TM^-based auto-segmentation. This denoising-based auto-segmentation method could provide more precise contour delineation of the liver and reduce the clinical workload.

**Abstract:**

Objective: This study aimed to investigate the segmentation accuracy of organs at risk (OARs) when denoised computed tomography (CT) images are used as input data for a deep-learning-based auto-segmentation framework. Methods: We used non-contrast enhanced planning CT scans from 40 patients with breast cancer. The heart, lungs, esophagus, spinal cord, and liver were manually delineated by two experienced radiation oncologists in a double-blind manner. The denoised CT images were used as input data for the AccuContour^TM^ segmentation software to increase the signal difference between structures of interest and unwanted noise in non-contrast CT. The accuracy of the segmentation was assessed using the Dice similarity coefficient (DSC), and the results were compared with those of conventional deep-learning-based auto-segmentation without denoising. Results: The average DSC outcomes were higher than 0.80 for all OARs except for the esophagus. AccuContour^TM^-based and denoising-based auto-segmentation demonstrated comparable performance for the lungs and spinal cord but showed limited performance for the esophagus. Denoising-based auto-segmentation for the liver was minimal but had statistically significantly better DSC than AccuContour^TM^-based auto-segmentation (*p* < 0.05). Conclusions: Denoising-based auto-segmentation demonstrated satisfactory performance in automatic liver segmentation from non-contrast enhanced CT scans. Further external validation studies with larger cohorts are needed to verify the usefulness of denoising-based auto-segmentation.

## 1. Introduction

In radiotherapy planning, organs at risk (OARs) are manually delineated by physicians based on computed tomography (CT) scans. Accurate contouring of OARs is essential for precise radiotherapy. OARs are manually delineated and carefully reviewed by physicians. However, it is a time-consuming process associated with an increased workload. The manual segmentation of OARs can take 1 h per patient due to the large number of axial slices. As such, atlas-based and deep-learning algorithms based on convolutional neural network auto-segmentation have been developed to alleviate the labor-intensive delineation of OARs [1,2,3,4,5,6,7]. Machine learning approaches, especially deep learning with multi-layered neural networks, have been actively applied to treatment planning in radiotherapy [8,9,10,11,12,13,14,15,16,17,18,19]. Many studies have investigated the deep-learning-based auto-segmentation of OARs for various disease sites [1,2,20,21,22,23,24,25,26,27]. The application of deep-learning algorithms based on convolutional neural networks has been proven effective and has shown high performance in delineating OARs [12,13,14,15,16,17,18,19,20,21,22]. Clinical practices use several commercially available deep-learning contouring products, including AccuContour^TM^ (Manteia Medical Technologies Co. Ltd., Xiamen, China).

Contrast-enhanced planning CT is used for the delineation of target volumes and OARs, and intravenous CT contrast can enhance normal tissue visualization and delineation [28,29]. Due to safety concerns associated with CT contrast, contrast-enhanced planning CT cannot be performed for all patients [30]. Therefore, non-contrast CT images have been used, but the boundary between the OARs and neighboring structures may be indistinguishable due to the suboptimal image quality of non-contrast CT [28,29]. In real-world clinical practice, portions of automatically generated contours in non-contrast CT require manual corrections to make them clinically acceptable. The noise in non-contrast CT images disturbs the visualization of structures, which increases the uncertainty of image segmentation. Deep-learning-based auto-segmentation algorithms have to overcome these image-related problems.

An image processing technique with improved performance, such as a denoising algorithm that can remove noise while maintaining the edge structure, is needed to achieve high-contrast CT images [31,32,33,34,35]. In a previous study, we implemented an anisotropic total variation (ATV) denoiser to enhance the image quality of low-dose cone-beam CT [36]. In this study, we establish that the segmentation accuracy can be improved significantly when this denoising technique is applied to increase the signal difference between structures of interest and unwanted noise in non-contrast CT. Moreover, we investigate the accuracy of segmentation when denoised non-contrast CT images are used as input images for deep-learning-based auto-segmentation of OARs. We compare the performance of this approach with manual segmentation and conventional deep-learning-based auto-segmentation without denoising.

## 2. Materials and Methods

### 2.1. Data and Delineation

Ethical approval for this study was obtained from the Institutional Review Board (IRB) of Yonsei University Health System, Gangnam Severance Hospital (Approval No.: 3-2021-0276). All methods were performed in accordance with the relevant guidelines and regulations. Due to the retrospective nature of this study, informed consent was waived by the IRB of Gangnam Severance Hospital. We used non-contrast planning CT scans of female patients with breast cancer who underwent modified radical mastectomy or breast-conserving surgery and received postoperative radiotherapy between 2019 and 2020 [37]. Forty patients were randomly chosen. The median age was 49 years old (range, 30–77 years), and the median body mass index was 22 kg/m^2^ (range 17–32 kg/m^2^). There were 22 patients with left breast cancer and 18 patients with right breast cancer. No patients had previously undergone surgical procedures for lung, heart, esophagus, spine, and upper abdominal organs at the time of conducting non-contrast planning CT. The CT images were acquired on a Siemens Sensation Open scanner (Siemens, Forchheim, Germany) using the following parameters: 120 kVp (scan voltage) and 3 mm slice thickness (layer thickness). Scans were conducted with tube current modulation, an adaptive method in which the current changes as the gantry is rotated. We obtained 81–123 slices per patient. All patients were scanned in the supine position with a customized arm support using a breast board. In this study, the OARs included the heart, right and left lungs, esophagus, spinal cord, and liver. The contours were manually delineated by two experienced radiation oncologists. The radiation oncologist was blinded to the results of delineation of the OARs by other radiation oncologists.

### 2.2. Deep-Learning-Based Auto-Segmentation

Recently, various deep-learning-based auto-segmentation methods have been developed to assist with image segmentation tasks. Satisfactory organ segmentation results have been reported [1], and some commercial products have been implemented in clinics for CT-based automatic segmentation. In this study, a commercially available deep learning contouring software “AccuContour^TM^” (Manteia Medical Technologies Co. Ltd., Xiamen, China) was used to generate the information required for treatment planning. It automatically segments the OARs, including the head-and-neck, thorax, abdomen, and pelvis for both males and females. AccuContour^TM^ is based on the U-net model [38] pre-trained by the vendors. U-net is a fully convolutional network (FCN) based model with end-to-end scheme proposed for image segmentation [38,39,40]. In U-net, the network for obtaining the overall context information of the image and that for accurate localization are symmetrically configured. U-net is a model that applies up-sampling and skip architecture of concepts that are more extended than FCNs, resulting in the U-net’s structure demonstrating superior performance in several image segmentation problems by leveraging data augmentation with only a small amount of learning data.

The trained model data were collected from multiple centers, and data cleaning was performed. Initial contours generated by the deep-learning model were corrected by post-processing with graph-based models. The accuracy was further improved by combining local and global information from the image and initial segmentation results. With these procedures, the contouring workload was reduced from hours to less than a minute for each patient. This segmentation technique was applied to delineate the OARs.

### 2.3. Anisotropic Total Variation Denoiser-Based Auto-Segmentation

An ATV denoiser [36] was applied to the CT images to augment the intensity difference between the striking features and unwanted noise by combining the conduction coefficient used in the anisotropic diffusion filter [41]. The minimization of the ATV objective function implies that edges with high contrast relative to the surroundings are preserved, and noisy voxels with low contrast are smoothed [42].

The ATV objective function, *R*(*V*), can be expressed as follows:

(1) $$R\left(V\right)=\underset{j}{\sum }R\left(V_{j}\right)=\underset{j}{\sum }w_{j}D\left(V_{j}\right)$$

where *w_j_* is the anisotropic penalty with different weights for neighbors at the same distance and *D*(*V_j_*) is the discrete gradient transform with backward difference in the *j*th indexed value of the CT images.

(2) $$D\left(V_{j}\right)=D\left(V_{\left(x,y\right)}\right)=\sqrt{{\left(V_{\left(x,y\right)}-V_{\left(x-1,y\right)}\right)}^{2}+{\left(V_{\left(x,y\right)}-V_{\left(x,y-1\right)}\right)}^{2}}$$

(3) $$w_{j}=\underset{m\in N_{j}}{\sum }exp\left[-{\left(\frac{V_{j}-V_{m}}{\delta }\right)}^{2}\right]$$

where index *j* identifies the index of voxel elements in the CT image, and $V_{\left(x,y\right)}$ is the voxel element at the 2D position (*x*, *y*). Equivalently, *N_j_* represents the set of neighbors of the *j*th voxel element. We only considered four first-order neighbors in this study. Empirically, the most meaningful results were derived with the parameter *δ* set to 80% of the cumulative distribution function histogram that accumulates the gradient at each voxel of a CT image.

The ATV objective function in Equation (1) was minimized using the steepest gradient descent method with an adaptive step size. It is expressed as follows.

(4) $$V_{j}^{t+1}=V_{j}^{t}-\lambda \nabla R\left(V_{j}\right)/\left|\nabla R\left(V\right)\right|$$

(5) $$\lambda =\gamma \sqrt{\underset{j}{\sum }{\left(V_{j}^{t}\right)}^{2}}$$

where *λ* is an adaptive parameter that reduces the smoothing degree as the iteration progresses [27,31]. The square root of all voxel elements updated in each step is used to change *λ* gradually to smaller values with an increase in the number of iterations. A scaling parameter γ was used to escape local minimization due to sudden changes. This value starts initially at 1.0 and decreases linearly by multiplication with a constant value (0.8) when R(V) in the current iteration step is greater than that in the preceding step. $\nabla R\left(V_{j}\right)$ is the gradient of the objective function R(V) at the *j*th indexed pixel [32]. The root-sum-square of the gradient calculated at all the pixels, |∇R(V)|, is required for the normalized gradient calculation [32]. The number of iterations is fine-tuned for the gradient descent optimizer. In this study, the optimal number of iterations was set to 20. The parameters used to optimize the denoising method were based on the manuaaly adjusted analysis. The pseudo-code of the ATV denoiser is presented in Appendix A.

**Figure 1:**
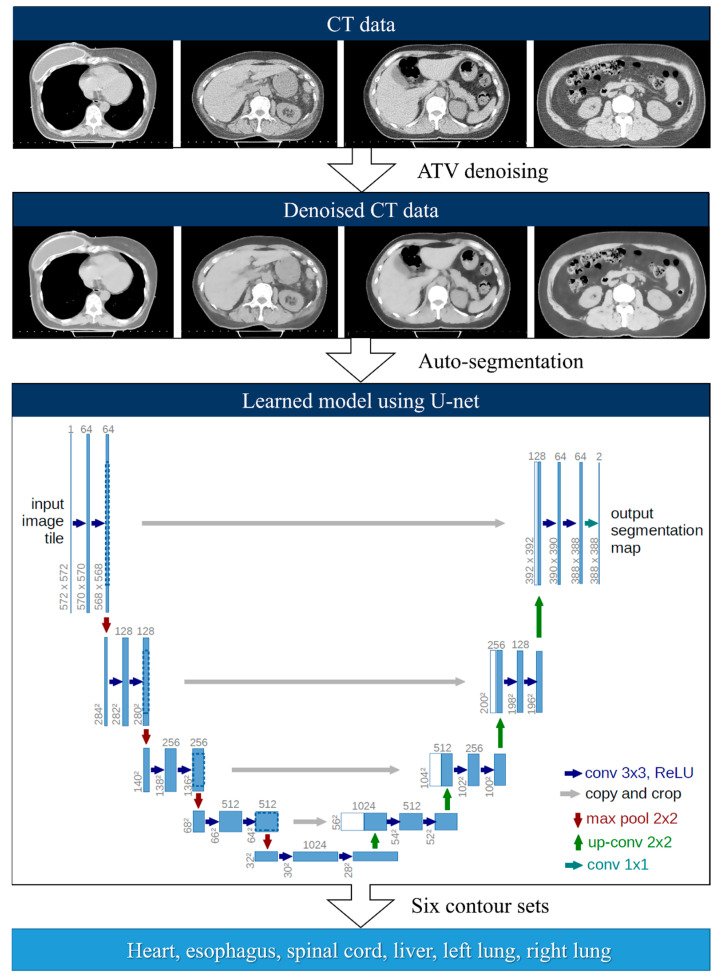
Deep-learning-based auto-segmentation framework.

The proposed image processing pipeline includes three steps. The first step is denoising. The anisotropic total variation was used for each image set to smooth noisy pixels while preserving the intensity of the edges during denoising. For the second step, the denoised CT images were used as input data in the AccuContour^TM^ segmentation module based on the U-net model pre-trained by the vendors. The third step involves contour segmentation. Six auto-generated contour sets (heart, left lung, right lung, esophagus, spinal cord, and liver) for each CT image were generated using a deep-learning-based auto-segmentation framework (Figure 1).

### 2.4. Quantitative Analysis

The noise power spectrum (NPS) was calculated using open-source software (imQuest, Duke University, Durham, NC, USA) that uses the technology described in TG233 of the AAPM [43,44] to assess the image quality characteristics without and with ATV denoiser. The quantity of in-plane noise was evaluated using two-dimensional NPS. For CT images, NPS can be determined in structures with a homogenous area. In this study, the liver was selected for NPS calculations, and overall frequencies were compared using 1D profiles.

The manual contours drawn by two radiation oncologists were considered the ground truth in this study, against which the AccuContour^TM^-based and denoising-based auto-segmentations were compared. To quantitatively evaluate the accuracy of AccuContour^TM^-based and denoising-based auto-segmentations, the Dice similarity coefficient (DSC) was used to evaluate the performance of the proposed method. The DSC method calculates the overlapping results of two different volumes according to the following equation:

(6) $$\mathrm{DSC}=\frac{2\left|A\cap B\right|}{\left|A\right|+\left|B\right|}$$

where *A* is the manual segmentation volume, and *B* is the auto-segmentation volume (AccuContour^TM^ and denoising). DSC is a measure of overlap between two contours, from “0” to “1,” where “1” indicates a complete overlap. We considered a Dice score of 0.80 as an acceptable match [45].

Wilcoxon matched pairs signed-rank test was conducted, and statistical significance was defined as *p* < 0.05 for evaluating differences in the results of the DSC.

## 3. Results

**Figure 2:**
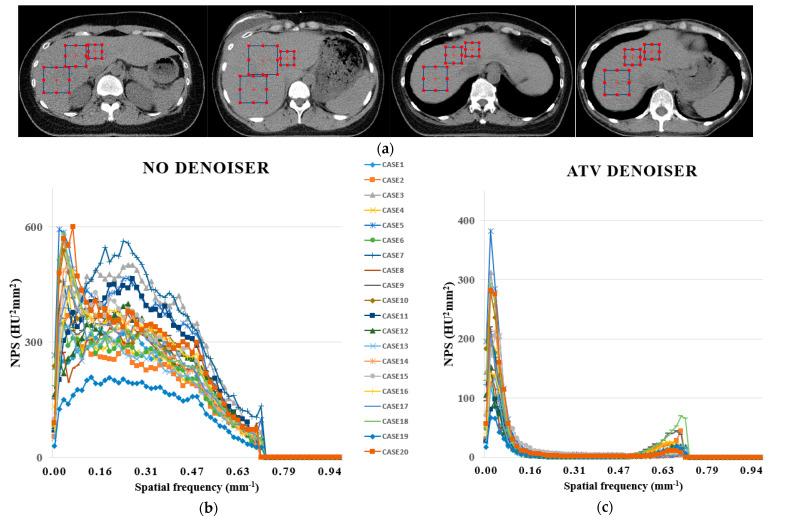

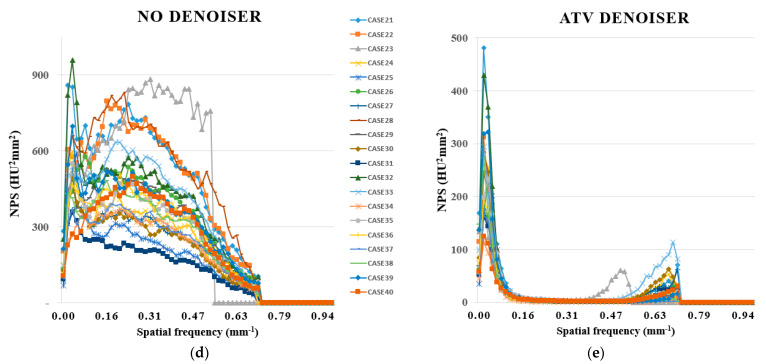
Noise power spectrum (NPS) for evaluating the noise texture and magnitude. (**a**) Examples of ROIs selected for NPS calculation on each slice. NPS curves using 40 patient data: (**b**) 1 to 20 cases without denoiser, (**c**) 1 to 20 cases with ATV, (**d**) 21 to 40 cases without denoiser, and (**e**) 21 to 40 cases with ATV.

Figure 2 shows the NPS curves without and with the ATV denoiser using planning CT images from 40 patients with breast cancer. Three square ROIs were placed at different positions in the liver area with uniform magnitude, as shown in Figure 2a. The ROIs were extended to five adjacent consecutive slices contained within the liver area. The average NPS peak frequency was obtained as 0.127 mm^−1^ without denoiser and 0.035 mm^−1^ for the ATV denoiser. The NPS peaks ranged from 209 to 957 HU^2^ mm^2^ without the denoiser and 66 to 481 HU^2^ mm^2^ for the ATV denoiser. As such, the NPS peak was on average lower with the ATV denoiser than without the denoiser. The peak spatial frequency values of NPS for ATV denoiser shifted to lower spatial frequencies in comparison to no denoiser. Numerically, the NPS average spatial frequencies were obtained as 0.142 mm^−1^ for the ATV denoiser and 0.295 mm^−1^ for no denoiser. Images with the ATV denoiser smoothed out with a lower noise amplitude, as indicated by the average frequencies of the NPS curves, resulting in a monotonous texture.

The results of DSC versus the manual contours from radiation oncologists 1 and 2 are shown in Table 1 and Table 2, respectively. The average DSC outcomes were higher than 0.80 in all OARs, except for the esophagus. The AccuContour^TM^-based and denoising-based auto-segmentations of the esophagus were below acceptable standards. In a comparison of AccuContour^TM^-based and denoising-based auto-segmentation, the differences were not statistically significant for the lungs, esophagus, or spinal cord (*p* > 0.05). The denoising-based auto-segmentations achieve superior segmentation accuracy on the liver and inferior segmentation accuracy on the heart compared with AccuContour^TM^-based auto-segmentations (*p* < 0.05).

## 4. Discussion

In this study, we compared the auto-contouring results in five organ structures using the commercial deep-learning contouring program AccuContour^TM^ with those obtained from an anisotropic total variation denoiser. Both the AccuContour^TM^-based and denoising-based auto-segmentation were considered to yield an acceptable accuracy for generating contours of the heart, lungs, spinal cord, and liver. However, these techniques yielded limited performance for the esophagus.

**Figure 3:**
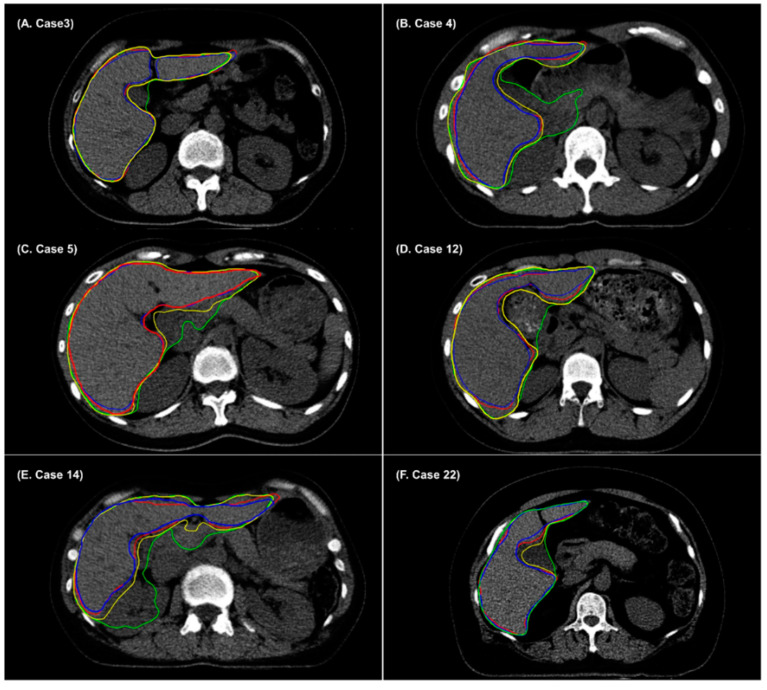
Example cases showing radiation oncologist 1’s manual contour (red), radiation oncologist 2’s manual contour (blue), AccuContour^TM^-based auto-segmentation (green), and denoising-based auto-segmentation (yellow) for the liver. AccuContour^TM^-based auto-segmentation over-contoured (**A**) gallbladder, (**B**) pancreas and portal vein, (**C**) pancreas and duodenum, (**D**) duodenum, (**E**) pancreas, duodenum, and kidney, and (**F**) gallbladder.

Deep-learning algorithms based on convolutional neural networks and AccuContour^TM^ have yielded satisfactory performance outcomes for the automatic segmentation of OARs. However, some parts of automatic segmentation of the liver in non-contrast CT required manual corrections to make them clinically acceptable (Figure 3). In non-contrast CT images, it might be difficult to delineate the fuzzy boundaries between the liver and adjacent organs, owing to low soft tissue contrast between the liver and its surrounding organs. In this study, the auto-segmentation results showed a significant improvement in the DSC when using denoising-based auto-segmentations of the liver, compared to using AccuContour^TM^ -based auto-segmentation. An ATV denoiser could enhance the image quality of CT by removing noisy areas, and this may lead to improved segmentation boundaries. Figure 3 shows that some parts surrounding the gall bladder, pancreas, duodenum, large vessel, or kidney are included in AccuContour^TM^-based auto-segmentation of the liver. However, denoising-based auto-segmentations could delineate the liver accurately by distinguishing the surrounding organs of CT images. These results indicate that the performance of denoising-based auto-segmentation is superior to that of AccuContour^TM^-based auto-segmentation. All deep-learning-based auto-segmentations should be carefully reviewed and approved by the radiation oncologists before use for a treatment plan. In some CT slices, major or minor errors of deep-learning-based auto-contour are present, and correction is required. The denoising-based auto-segmentation might convert “large or minor errors” of conventional deep-learning-based auto-segmentation to “minor errors” (a small amount of editing needed) or “no correction”. The denoising-based auto-segmentation could be a practical tool for reducing the clinical workload of radiotherapy planning.

**Figure 4:**
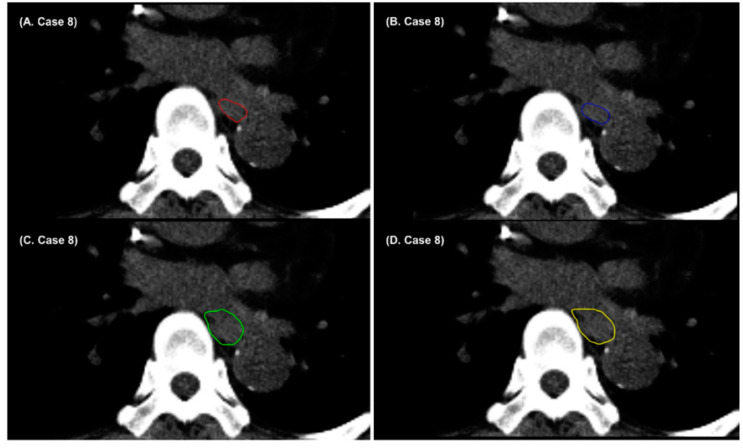
Example cases showing (**A**) radiation oncologist 1’s manual contour (red), (**B**) radiation oncologist 2’s manual contour (blue), (**C**) AccuContour^TM^-based auto-segmentation (green), and (**D**) denoising-based auto-segmentation (yellow) when the boundaries between the esophagus and surrounding soft tissues are not well-defined.

**Figure 5:**
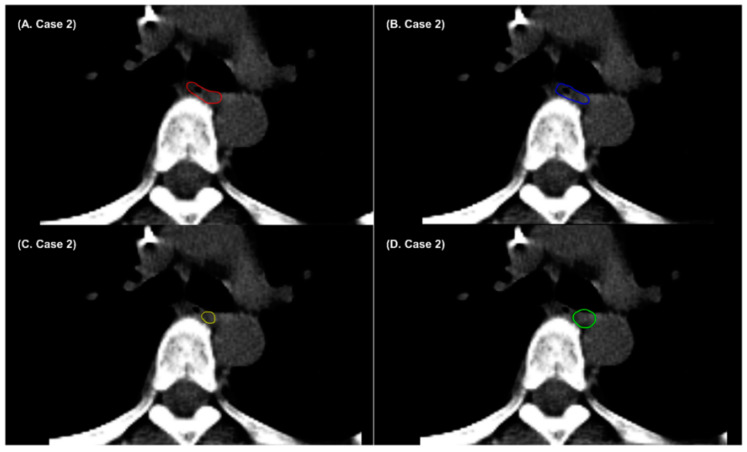
Example cases showing (**A**) radiation oncologist 1’s manual contour (red), (**B**) radiation oncologist 2’s manual contour (blue), (**C**) denoising-based auto-segmentation (yellow), and (**D**) AccuContour^TM^-based auto-segmentation (green) for the esophagus.

The esophagus is one of the most challenging OARs in thoracic organ auto-segmentation. In this study, the performance of AccuContour^TM^-based and denoising-based auto-segmentation was below a satisfactory level for the esophagus. Previous studies have reported that the DSCs of deep-learning-based auto-segmentation do not exceed 0.8 for the esophagus [40,46,47,48,49]. Due to the absence of a consistent intensity contrast between the esophagus and neighboring tissues in non-contrast CT images, the boundaries between the esophagus and surrounding soft tissues are not well-defined. Figure 4 shows that some parts of the surrounding pulmonary vessel were included in AccuContour^TM^-based and denoising-based auto-segmentation of the esophagus. In addition, the appearance of the esophagus varies depending on whether it is filled with air or not. Figure 5 shows that the air-filled regions of the esophagus were not included in AccuContour^TM^-based and denoising-based auto-segmentations. The segmentation results for the esophagus obtained from the denoising-based auto-segmentation may still be inaccurate and unsatisfactory.

**Figure 6:**
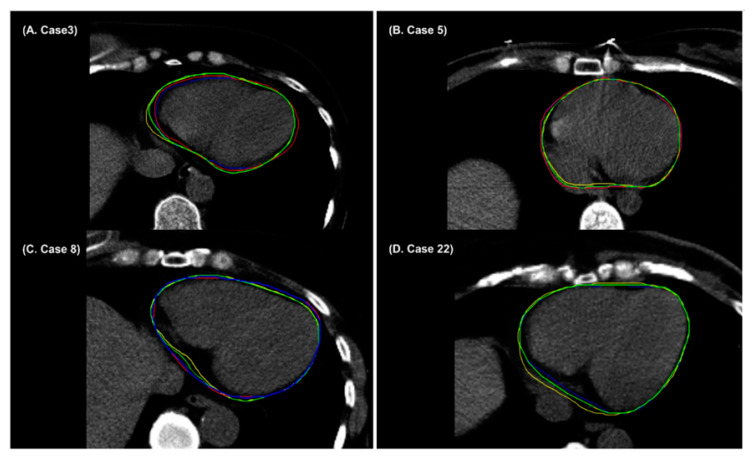
Example cases (**A**–**D**) showing radiation oncologist 1’s manual contour (red), radiation oncologist 2’s manual contour (blue), AccuContour^TM^-based auto-segmentation (green), and denoising-based auto-segmentation (yellow) for the heart.

It has been demonstrated that auto-segmentations for the heart and lungs yield higher DSCs, with an average of over 0.9 [45,48,49,50,51]. This study also showed that the average DSC outcomes of the heart and lungs are higher than 0.9. High-contrast edges and distinct structural boundaries of the heart and lungs were detected easily in both the AccuContour^TM^-based and denoising-based auto-segmentation. Therefore, AccuContour^TM^-based or denoising-based auto-segmentation for the heart and lungs can be used without major adjustments. The denoising-based auto-segmentations achieve inferior segmentation accuracy on the heart compared with AccuContour^TM^ -based auto-segmentations. The denoising-based auto-segmentation volumes are slightly larger than manual contours (Figure 6A,D) or smaller than manual contours (Figure 6B,C). Denoising-based auto-segmentation did not improve the accuracy of auto-segmentation of the heart.

This study had several limitations. Although 40 patients were randomly selected, selection bias in terms of the CT samples may be present. Since the results of this study were generated by only one proprietary software and CT scan, selection bias may have impacted our results.

Further, the contouring bias of the physicians may impact our results. Therefore, further external validation studies involving multiple experts, hospitals, and a larger sample size are needed to overcome these limitations. Moreover, denoising-based auto-segmentation should be compared using software other than AccuContour^TM^.

However, in several deep-learning-based automatic segmentation studies, a single experienced radiation oncologist delineated the organ at risk or the clinical target volume [10,11,14,50]. In this study, two radiation oncologists delineated the organ at risk because it was thought that inter-observer variability may exist. The results of the Dice similarity coefficient of the five organs at risk from radiation oncologist 1 and 2 were consistent, so the results of this study are expected to be relatively reliable.

## 5. Conclusions

CT images are subject to noise that can affect the boundary between the adjacent organs, providing potentially limited contrast. Reducing the noise level in CT images provides the best visualization of structures, which increases the accuracy of image segmentation.

By combining the denoising algorithm in the deep-learning based auto-segmentation, the denoising-based auto-segmentation results of the liver from non-contrast CT scans were slightly superior to those of commercial conventional deep-learning-based auto-segmentation and had greater similarities with the ground truth. This denoising-based auto-segmentation could provide a more precise contour delineation of the liver, thus reducing the clinical workload. The results of this study require validation through further studies using a higher sample size, which will compare denoising-based auto-segmentation using software other than AccuContour^TM^.

## Figures and Tables

**Table 1:** Comparison of the dice similarity coefficients (DSC) results generated from AccuContour^TM^ and denoising-based auto-segmentations using the radiation oncologist 1’s manual contours as reference.

Case	Heart		Right Lung		Left Lung		Esophagus		Spinal Cord		Liver	
	* Manteia	† Denoiser	Manteia	Denoiser	Manteia	Denoiser	Manteia	Denoiser	Manteia	Denoiser	Manteia	Denoiser
Case 1	0.964	0.960	0.982	0.983	0.981	0.981	0.685	0.742	0.777	0.824	0.953	0.954
Case 2	0.951	0.947	0.980	0.980	0.979	0.979	0.670	0.723	0.891	0.873	0.936	0.939
Case 3	0.958	0.952	0.982	0.982	0.979	0.979	0.660	0.602	0.881	0.884	0.957	0.963
Case 4	0.931	0.928	0.971	0.970	0.975	0.975	0.744	0.719	0.866	0.858	0.937	0.938
Case 5	0.908	0.894	0.971	0.971	0.975	0.975	0.745	0.672	0.877	0.877	0.948	0.952
Case 6	0.930	0.929	0.982	0.982	0.973	0.974	0.573	0.691	0.877	0.877	0.890	0.926
Case 7	0.978	0.977	0.951	0.951	0.954	0.955	0.735	0.730	0.876	0.870	0.874	0.864
Case 8	0.929	0.926	0.971	0.972	0.970	0.970	0.669	0.662	0.859	0.856	0.964	0.963
Case 9	0.938	0.936	0.978	0.978	0.972	0.972	0.664	0.732	0.814	0.819	0.959	0.960
Case 10	0.952	0.947	0.982	0.982	0.981	0.981	0.765	0.786	0.883	0.877	0.959	0.963
Case 11	0.945	0.944	0.979	0.979	0.977	0.978	0.746	0.737	0.838	0.831	0.920	0.924
Case 12	0.946	0.941	0.962	0.963	0.964	0.966	0.680	0.634	0.868	0.848	0.933	0.933
Case 13	0.952	0.950	0.984	0.984	0.981	0.982	0.681	0.695	0.865	0.854	0.926	0.932
Case 14	0.934	0.930	0.981	0.981	0.981	0.981	0.570	0.633	0.858	0.854	0.909	0.933
Case 15	0.930	0.926	0.975	0.976	0.973	0.973	0.702	0.744	0.859	0.865	0.893	0.931
Case 16	0.906	0.898	0.934	0.934	0.940	0.940	0.691	0.637	0.870	0.870	0.956	0.959
Case 17	0.939	0.932	0.977	0.977	0.972	0.972	0.697	0.697	0.857	0.863	0.954	0.955
Case 18	0.950	0.948	0.976	0.976	0.979	0.979	0.725	0.697	0.878	0.882	0.939	0.942
Case 19	0.910	0.887	0.981	0.981	0.981	0.979	0.653	0.617	0.853	0.859	0.873	0.885
Case 20	0.877	0.875	0.983	0.984	0.975	0.975	0.730	0.775	0.853	0.846	0.954	0.956
Case 21	0.919	0.921	0.981	0.981	0.978	0.978	0.727	0.719	0.819	0.801	0.940	0.941
Case 22	0.999	0.968	0.998	0.992	0.998	0.992	0.990	0.797	0.805	0.807	0.935	0.951
Case 23	0.956	0.954	0.977	0.977	0.980	0.979	0.625	0.636	0.878	0.872	0.954	0.957
Case 24	0.963	0.962	0.986	0.986	0.979	0.980	0.728	0.725	0.872	0.857	0.955	0.956
Case 25	0.919	0.920	0.987	0.987	0.979	0.978	0.767	0.763	0.872	0.874	0.964	0.967
Case 26	0.966	0.966	0.990	0.989	0.986	0.987	0.813	0.821	0.892	0.876	0.964	0.966
Case 27	0.963	0.961	0.992	0.992	0.989	0.989	0.650	0.638	0.797	0.815	0.959	0.960
Case 28	0.963	0.963	0.991	0.991	0.984	0.985	0.785	0.789	0.853	0.861	0.959	0.960
Case 29	0.967	0.967	0.985	0.985	0.976	0.978	0.765	0.768	0.823	0.836	0.934	0.937
Case 30	0.951	0.951	0.985	0.985	0.980	0.980	0.661	0.671	0.818	0.835	0.927	0.930
Case 31	0.950	0.950	0.986	0.986	0.984	0.984	0.668	0.650	0.841	0.844	0.896	0.899
Case 32	0.947	0.946	0.986	0.986	0.980	0.980	0.791	0.778	0.865	0.863	0.911	0.910
Case 33	0.956	0.956	0.962	0.962	0.975	0.975	0.771	0.780	0.831	0.846	0.948	0.950
Case 34	0.950	0.950	0.978	0.978	0.979	0.980	0.740	0.725	0.861	0.857	0.943	0.946
Case 35	0.947	0.949	0.977	0.977	0.981	0.981	0.749	0.739	0.887	0.880	0.956	0.959
Case 36	0.937	0.938	0.985	0.985	0.977	0.978	0.788	0.782	0.881	0.883	0.950	0.952
Case 37	0.945	0.945	0.984	0.984	0.980	0.981	0.730	0.727	0.879	0.882	0.939	0.938
Case 38	0.933	0.934	0.987	0.987	0.983	0.982	0.809	0.819	0.872	0.878	0.947	0.949
Case 39	0.945	0.945	0.968	0.962	0.974	0.975	0.759	0.743	0.887	0.894	0.952	0.953
Case 40	0.911	0.911	0.982	0.982	0.977	0.978	0.664	0.672	0.876	0.868	0.947	0.947
Average	0.943	0.940	0.979	0.978	0.977	0.977	0.719	0.717	0.858	0.868	0.938	0.943
*p*-value	0.000		0.091		0.095		0.705		0.762		0.000	

* AccuContour^TM^ (Manteia Medical Technologies Co. Ltd., Xiamen, China)-based auto-segmentation, † Denoising-based auto-segmentation.

**Table 2:** Comparison of the dice similarity coefficients (DSC) results generated from AccuContour^TM^ and denoising-based auto-segmentations using the radiation oncologist 2’s manual contours as reference.

Case	Heart		Right Lung		Left Lung		Esophagus		Spinal Cord		Liver	
	* Manteia	† Denoiser	Manteia	Denoiser	Manteia	Denoiser	Manteia	Denoiser	Manteia	Denoiser	Manteia	Denoiser
Case 1	0.964	0.960	0.981	0.981	0.980	0.980	0.685	0.742	0.749	0.785	0.953	0.955
Case 2	0.937	0.934	0.985	0.985	0.982	0.982	0.696	0.730	0.872	0.844	0.932	0.934
Case 3	0.964	0.962	0.984	0.984	0.980	0.981	0.638	0.597	0.865	0.867	0.949	0.956
Case 4	0.912	0.909	0.973	0.972	0.979	0.979	0.741	0.697	0.857	0.864	0.927	0.929
Case 5	0.907	0.895	0.977	0.977	0.977	0.976	0.721	0.625	0.848	0.848	0.941	0.947
Case 6	0.927	0.927	0.987	0.987	0.980	0.980	0.577	0.693	0.887	0.892	0.884	0.920
Case 7	0.986	0.989	0.996	0.997	0.995	0.996	0.808	0.839	0.870	0.873	0.849	0.859
Case 8	0.925	0.922	0.964	0.963	0.973	0.973	0.658	0.689	0.872	0.851	0.954	0.953
Case 9	0.934	0.933	0.979	0.978	0.974	0.974	0.702	0.747	0.851	0.852	0.952	0.952
Case 10	0.916	0.909	0.984	0.984	0.984	0.983	0.732	0.756	0.869	0.852	0.957	0.959
Case 11	0.938	0.939	0.986	0.986	0.974	0.974	0.683	0.683	0.786	0.749	0.912	0.915
Case 12	0.949	0.943	0.977	0.977	0.967	0.969	0.707	0.655	0.778	0.747	0.925	0.925
Case 13	0.935	0.935	0.985	0.985	0.982	0.982	0.665	0.692	0.844	0.823	0.921	0.925
Case 14	0.935	0.933	0.984	0.984	0.979	0.979	0.633	0.695	0.823	0.790	0.902	0.927
Case 15	0.907	0.909	0.983	0.983	0.976	0.976	0.660	0.648	0.835	0.841	0.921	0.880
Case 16	0.926	0.936	0.985	0.985	0.979	0.979	0.681	0.637	0.781	0.787	0.953	0.956
Case 17	0.939	0.932	0.977	0.977	0.972	0.972	0.697	0.697	0.804	0.822	0.949	0.949
Case 18	0.950	0.951	0.982	0.982	0.982	0.982	0.734	0.755	0.870	0.880	0.932	0.936
Case 19	0.890	0.867	0.985	0.985	0.983	0.981	0.630	0.604	0.860	0.865	0.857	0.869
Case 20	0.850	0.848	0.986	0.986	0.979	0.978	0.738	0.743	0.885	0.860	0.949	0.950
Case 21	0.954	0.955	0.991	0.991	0.986	0.986	0.730	0.731	0.853	0.835	0.946	0.947
Case 22	0.999	0.968	0.995	0.990	0.995	0.989	0.902	0.752	0.790	0.827	0.941	0.952
Case 23	0.956	0.954	0.975	0.975	0.978	0.978	0.622	0.632	0.838	0.816	0.953	0.955
Case 24	0.963	0.962	0.984	0.984	0.977	0.978	0.726	0.724	0.838	0.820	0.956	0.958
Case 25	0.924	0.925	0.986	0.985	0.978	0.980	0.767	0.763	0.860	0.864	0.962	0.965
Case 26	0.966	0.966	0.986	0.986	0.983	0.984	0.813	0.821	0.868	0.861	0.960	0.961
Case 27	0.963	0.961	0.990	0.990	0.987	0.988	0.636	0.627	0.840	0.837	0.959	0.959
Case 28	0.963	0.963	0.991	0.991	0.984	0.985	0.785	0.790	0.853	0.862	0.951	0.951
Case 29	0.967	0.967	0.984	0.983	0.974	0.976	0.765	0.768	0.847	0.827	0.934	0.936
Case 30	0.951	0.951	0.984	0.984	0.979	0.980	0.661	0.671	0.857	0.840	0.938	0.939
Case 31	0.932	0.932	0.986	0.986	0.983	0.983	0.668	0.650	0.852	0.843	0.895	0.897
Case 32	0.939	0.938	0.985	0.985	0.978	0.978	0.791	0.778	0.857	0.855	0.912	0.911
Case 33	0.956	0.956	0.960	0.960	0.975	0.975	0.710	0.709	0.857	0.839	0.948	0.950
Case 34	0.949	0.948	0.976	0.976	0.976	0.977	0.739	0.723	0.876	0.861	0.947	0.949
Case 35	0.935	0.934	0.982	0.982	0.980	0.980	0.749	0.739	0.880	0.874	0.953	0.955
Case 36	0.936	0.936	0.984	0.984	0.976	0.976	0.793	0.786	0.860	0.840	0.951	0.952
Case 37	0.945	0.945	0.982	0.982	0.978	0.979	0.669	0.662	0.812	0.821	0.938	0.936
Case 38	0.944	0.945	0.986	0.986	0.981	0.981	0.809	0.819	0.870	0.874	0.950	0.951
Case 39	0.955	0.955	0.965	0.960	0.974	0.975	0.682	0.663	0.884	0.892	0.953	0.953
Case 40	0.911	0.911	0.980	0.980	0.977	0.977	0.624	0.630	0.874	0.875	0.948	0.948
Average	0.940	0.938	0.982	0.982	0.979	0.980	0.711	0.709	0.847	0.841	0.935	0.938
*p*-value	0.008		0.091		0.097		0.984		0.082		0.000	

* AccuContour^TM^ (Manteia Medical Technologies Co. Ltd., Xiamen, China)-based auto-segmentation, † Denoising-based auto-segmentation.

## Data Availability

All data generated or analyzed during this study are included in the article.

## Appendix A

For n←1 to all images do For j←1 to all voxels do $Calculate\;D\left(V_{j}\right)\;using\;Eq.\;\left(2\right)$ *Create gradient CDF histogram using*$D\left(V_{j}\right)$ *End For* δ←Value at 80% of gradient CDF *End For* For n←1 to all images do R(V)←0$,\;r\leftarrow 1,\;r_{red}\leftarrow 0.8$ For j←1 to all voxels do $w_{j}\leftarrow 0$ $For\;m\leftarrow 1\;to\;N_{j}\;do$ $w_{j}\leftarrow w_{j}+exp\left[-{\left(\frac{V_{j}-V_{m}}{\delta }\right)}^{2}\right]$ End For *Calculate*$D\left(V_{j}\right)$*using Equation (2)* $R\left(V_{j}\right)\leftarrow w_{j}D\left(V_{j}\right)$ $R\left(V\right)\leftarrow R\left(V\right)+R\left(V_{j}\right)$ *End For* For t←1 to 20 do |∇R(V)|←0 For j←1 to all voxels do $\lambda \leftarrow \sqrt{\underset{j}{\sum }V_{j}^{2}}$ λ←λ×r $\mathrm{Calculate}\;\nabla R\left(V_{j}\right)$ $\left|\nabla R\left(V\right)\right|\leftarrow \left|\nabla R\left(V\right)\right|+{\left(\nabla R\left(V_{j}\right)\right)}^{2}$ *End For* $\left|\nabla R\left(V\right)\right|\leftarrow \sqrt{{\left|\nabla R\left(V\right)\right|}^{2}}$ For j←1 to all voxels do $V_{j}^{\prime }\leftarrow V_{j}+\lambda \nabla R\left(V_{j}\right)/\left|\nabla R\left(V\right)\right|$ *Calculate*$D\left(V_{j}^{\prime }\right)$*using Equation (2)* *End For* *Calculate*$R\left(V^{\prime }\right)$*using*$w_{j}\;and\;D\left(V_{j}^{\prime }\right)$ *While R*($V_{j}^{\prime })>R\left(V\right)\;do$ $r\leftarrow r\times r_{red}$ λ←λ×r For j←1 to all voxels do $V_{j}^{\prime }\leftarrow V_{j}+\frac{\lambda \nabla R\left(V_{j}\right)}{\left|\nabla R\left(V\right)\right|}$ *Calculate*$D\left(V_{j}^{\prime }\right)$*using Equation (2)* *End For* *Calculate*$R\left(V^{\prime }\right)$ *End While* *Update*$V_{j}^{\prime }\;to\;V_{j}$ *End For* *End For*
